# Pediatric intranasal lobular capillary hemangioma: A case report

**DOI:** 10.1016/j.ijscr.2023.108894

**Published:** 2023-10-01

**Authors:** Asma Iqbal, Syed Faqeer Hussain Bokhari, Syed Anwar Ahmad

**Affiliations:** King Edward Medical University, Mayo Hospital, Lahore, Pakistan

**Keywords:** Pediatrics, LCH, Case report, Nasal cavity, Vascular lesion

## Abstract

**Introduction and importance:**

Lobular capillary hemangioma (LCH) is a benign vascular lesion typically affecting the head and neck region, especially the nasal cavity. However, its occurrence in the nasal cavity of the pediatric population is rare, making its diagnosis and management crucial in such cases.

**Case presentation:**

A 7-year-old female presented with left-sided nasal obstruction and recurrent epistaxis for 4 months. Examination revealed a dark purplish-red nasal mass, which bled easily upon probing. CT scans showed a soft tissue lesion with post-contrast enhancement. Histopathological examination confirmed lobular capillary hemangioma. The patient successfully underwent surgical excision without pre-operative embolization.

**Clinical discussion:**

LCH affects both the skin and mucous membranes. Although its exact etiology remains debated, trauma is a leading risk factor for its development. Biopsy and radiological investigations confirm its diagnosis. Differentiating LCH from other vascular disorders with similar presentations is essential for a thorough understanding and better management. Endoscopic surgical excision combined with electrodesiccation is the treatment of choice.

**Conclusion:**

Intranasal LCH in children is infrequent. An accurate diagnosis is essential for a comprehensive understanding. Surgical excision with electrodesiccation is the preferred treatment, but the role of pre-operative embolization is still under discussion.

## Highlights

• Lobular capillary **hemangioma** is a rare type of vascular tumor in the pediatric population that **primarily** affects the skin and mucous membranes.
• In females, it is mucosal in origin. While, in males, it is of cutaneous origin.
• The **exact** cause of intranasal capillary **hemangioma** has not been identified, but risk factors like trauma are major contributors.
• Lobular capillary **hemangioma** is characterized by pronounced lobules with large vascular spaces and endothelial proliferation.
• Radiological investigations help confirm the diagnosis and the primary treatment is surgical resection combined with **electrodesiccation.**

## 1 Introduction

Lobular capillary hemangioma (LCH) is a benign vascular lesion that commonly affects the head and neck region [1]. It primarily involves the skin and mucous membranes, with the nose being a frequently affected site [2]. It is very rare in pediatric population, especially in the nasal cavity [3]. LCH in the nasal cavity typically presents with symptoms such as epistaxis and nasal obstruction. The histopathological examination of LCH reveals distinct features, including extensive endothelial proliferation with dilated capillaries arranged in lobules. While the exact cause of LCH is not fully understood, certain factors such as a history of trauma and pregnancy have been associated with its development [4]. Surgical endoscopic excision with complete electrodesiccation of the tumor base is considered the preferred treatment approach [5].

In this case report, we present the clinical findings and management of a 7-year-old female patient who presented with left-sided nasal obstruction and recurrent episodes of epistaxis. The patient had a history of frequent nasal picking, but there were no other significant medical or surgical factors. Upon examination, a dark purplish-red nasal mass was identified on the left side, which exhibited granular consistency and bled easily upon probing. Imaging studies, including a contrast-enhanced CT of the nose and paranasal sinuses, revealed a rounded soft tissue density lesion originating from the anterior floor of the nose. Histopathological analysis of the biopsy confirmed the diagnosis of lobular capillary hemangioma. The patient underwent complete excision of the mass under general anesthesia without pre-operative embolization. Postoperatively, the patient experienced an uneventful recovery and showed no recurrence of the hemangioma during the 3-month follow-up period.

## 2 Case report

**Fig. 1:**
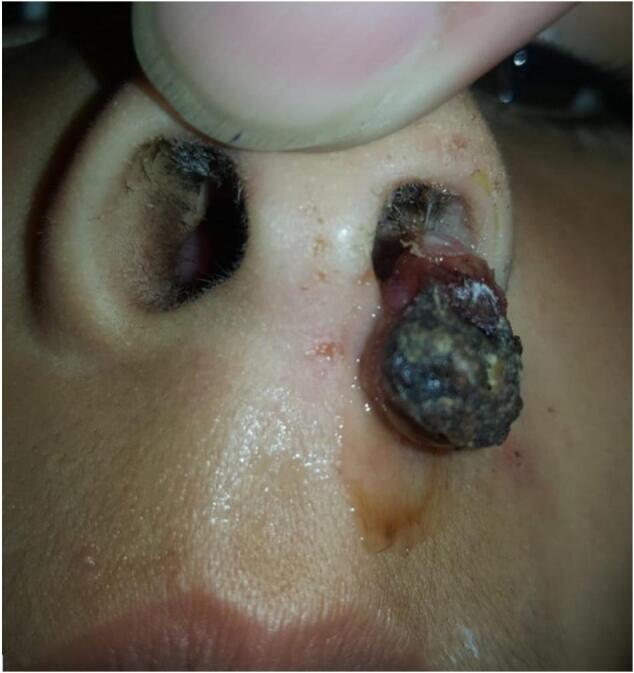
A purplish-red nasal mass (2 × 1) cm extending from the floor of the nose.

A 7-year-old female presented to the ENT department through the outpatient department complaining of left-sided nasal obstruction and frequent episodes of left-sided epistaxis for the past 4 months. There were no associated symptoms. Past medical and surgical history was insignificant. Traumatic history included frequent nasal picking. Family history was insignificant for bleeding disorders or malignancy. Examination of the nasal cavity revealed a left-sided nasal mass. It was dark purplish-red in color, granular in consistency, and bled readily on probing (Fig. 1).

**Fig. 2:**
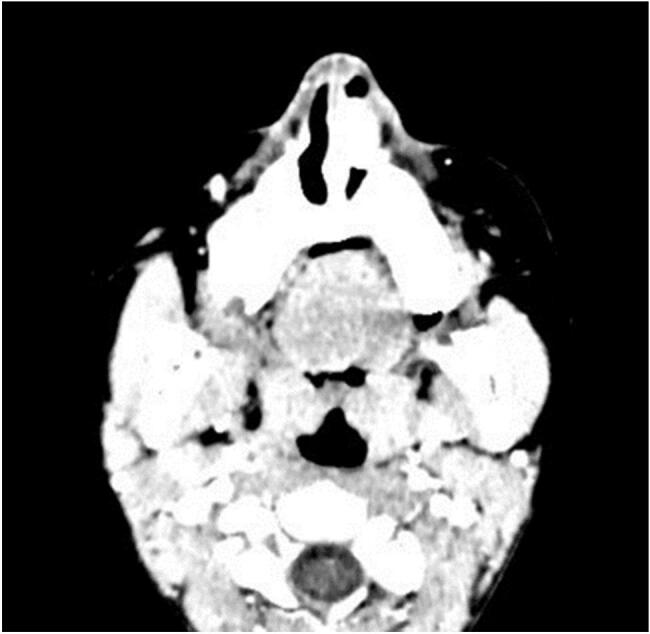
CT nose and paranasal sinuses showed rounded soft tissue hyperdense lesion arising from the left side of floor of the anterior portion of the nose with intense post contrast enhancement with density of 275 HU (Axial Cut).

**Fig. 3:**
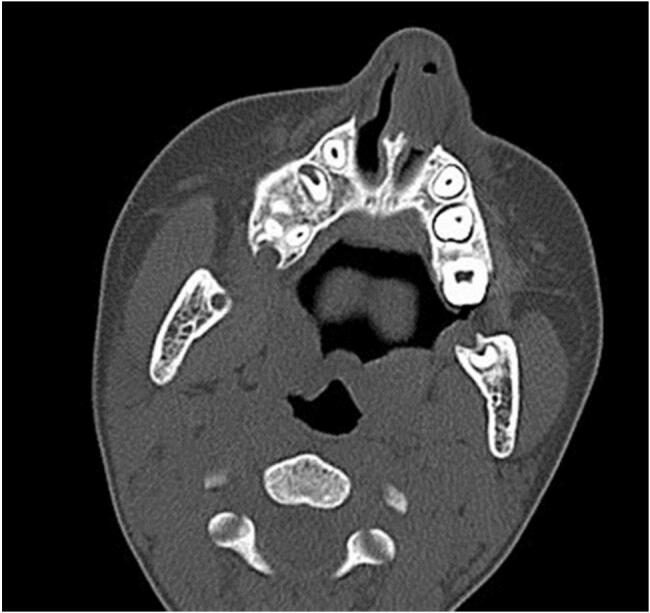
CT-scan (Axial Cut) bony window showed opacity in the left side of the nasal cavity without causing any bone erosion.

The point of attachment of the pedicle could not be assessed due to bleeding and irritability. There were no other significant findings on history and examination. Laboratory examinations were also insignificant. CT nose and paranasal sinuses with contrast were performed. A rounded soft tissue density lesion was seen arising from the left side of the floor of the anterior portion of the nose with an intense post-contrast enhancement density of 275 HU (Fig. 2). There were no signs of bone erosion (Fig. 3). A biopsy of the mass was sent for histopathological analysis which revealed lobular capillary haemangioma. The mass was completely excised without pre-operative embolization under general anesthesia and the pedicle was cauterized using bipolar diathermy. Anterior nasal packing was placed for 24 h. The postoperative period was uneventful. After 24 h, the nasal packing was removed and the patient was discharged. She was followed up for 3 months and showed a healed mucosa with no recurrence of the hemangioma.

## 3 Discussion

Lobular capillary hemangioma (LCH), formerly known as pyogenic granuloma, is a benign vascular proliferation of idiopathic etiology [6]. It commonly affects the mucous membranes of the oral cavity as well as the skin of the head and neck area. LCH in the nasal cavity is rare, especially in young children. In children, capillary hemangiomas make up about 7 % of all benign tumors in the head and neck, with the most common site being the lip (38 %) followed by the nasal cavity (29 %), oral mucosa (18 %), and tongue (15 %) [7,8].

LCH can present as either a mucosal variant or a cutaneous variant. The mucosal variant is more frequently seen in women, while the cutaneous variant is more common in men [9]. The mucosal variant of LCH primarily occurs in specific areas of the nasal cavity, including the anterior nasal septum (Little's area), the inferior turbinate, and the vestibule. These regions are more vulnerable to local trauma, which is believed to play a role in the development of LCH.

Trauma, such as recurrent nose picking or a history of nasal packing, has been associated with LCH in the anterior nasal septum (Kiesselbach's area) [4]. However, studies have shown that only a small percentage of patients with clinically diagnosed LCH have a history of trauma [10]. Therefore, the exact cause and origin of LCH remain unknown.

Since various benign and malignant tumors might appear with identical symptoms, thorough investigation is necessary to determine the diagnosis of LCH. Some benign lesions that need to be distinguished from LCH are Wegener's granulomatosis, meningoencephalocele, sarcoidosis, nasal polyps, gliomas, hemangiomas, lipomas, osteomas, fibromas, nasopharyngeal cysts, and histiocytomas. Angiosarcoma, aesthesioneuroblastoma, squamous cell carcinoma, achromic melanoma, Kaposi sarcoma, and adenocarcinoma lymphoma are among the malignant lesions that need to be ruled out [[11], [12], [13]].

Radiological studies, such as contrast-enhanced computed tomography (CT) and magnetic resonance imaging (MRI), are commonly used to evaluate intranasal masses. CT scans typically show a well-defined, strongly enhancing soft tissue mass in cases of LCH [14,15]. MRI reveals hypointensity and hyperintensity in T1 and T2 weighted images, respectively [16]. Bony destruction in LCH is rare but can occur due to the compressive devascularisation effect of the mass.

Microscopic examination of the affected tissue is necessary for a definitive diagnosis of LCH. Histopathologically, LCH is characterized by extensive endothelial proliferation with prominent vascular spaces, capillaries arranged in a lobular pattern, epithelial ulceration, and a fibrovascular tissue base [17].

The primary treatment for intranasal LCH is endoscopic surgical excision, combined with complete electrodesiccation of the tumor base [18]. In some cases, pre-operative embolization may be used to achieve vascular control before surgery [2]. However, there is debate regarding the necessity of pre-operative embolization, and some experts argue that surgery alone is sufficient [19]. Resection of the lesion is typically performed along the subperichondral or subperiosteal plane, with a margin of healthy mucosa. Recurrence rates vary among studies, with some reporting no recurrence and others reporting a rate of 42 %, but LCH does not have the potential for malignant transformation [5,20].

## 4 Conclusions

LCH in the nasal cavity is a rare condition, more commonly found in women between the third and fifth decades of life and rare in pediatric population. It presents as a benign vascular proliferation with a lobular architecture. Diagnosis requires careful evaluation to differentiate it from other benign and malignant lesions. Endoscopic surgical excision with complete electrodesiccation is the preferred treatment, although the need for pre-operative embolization remains controversial. Histopathological examination confirms the diagnosis of LCH, and long-term follow-up is essential to monitor for recurrence.

## Ethical approval

As it's a case report, it is exempted from ethical approval by the Institutional Board of Review, King Edward Medical University, Lahore.

## Funding

None.

## Author contribution

**Asma Iqbal**: Study conception & Design

**Syed Faqeer Hussain Bokhari**: Writing of article

**Syed Anwar Ahmad**: Data acquisition

## Guarantor

**Syed Faqeer Hussain Bokhari:** MBBS, King Edward Medical University, Lahore. (drfhbokhari512@gmail.com)

## Research registration number

N/A

## Informed consent

Written informed consent was obtained from the patient's parents/legal guardian for publication and any accompanying images. A copy of the written consent is available for review by the Editor-in-Chief of this journal on request.

## Methods

The work has been reported in line with the SCARE criteria [21].

## Conflict of interest statement

None to disclose.

## References

[1] Albesher M.B., Alharbi M.H., Alsumairi M.B., Hussein N.M. (2022). Nasal lobular capillary hemangioma: report of a case managed by endoscopic excision and pre-operative angio-embolization. Int. J. Surg. Case Rep..

[2] Tamaki A., Babajanian E., D’Anza B., Rodriguez K. (2017). Lobular capillary hemangiomas: case report and review of literature of vascular lesions of the nasal cavity. Am. J. Otolaryngol..

[3] Mariño-Sánchez F., Lopez-Chacon M., Jou C., Haag O. (2016). Pediatric intranasal lobular capillary hemangioma: report of two new cases and review of the literature. Respir. Med. Case. Rep..

[4] Puxeddu R., Berlucchi M., Ledda G.P., Parodo G., Farina D., Nicolai P. (2006). Lobular capillary hemangioma of the nasal cavity: a retrospective study on 40 patients. Am. J. Rhinol..

[5] Smith S.C., Patel R.M., Lucas D.R., McHugh J.B. (2013). Sinonasal lobular capillary hemangioma: a clinicopathologic study of 34 cases characterizing potential for local recurrence. Head Neck Pathol..

[6] Patrice S.J., Wiss K., Mulliken J.B. (1991). Pyogenic granuloma (lobular capillary hemangioma): a clinicopathologic study of 178 cases. Pediatr. Dermatol..

[7] Leyman B., Govaerts D., Dormaar J.T. (2023). A 16-year retrospective study of vascular anomalies in the head and neck region. Head Face Med..

[8] Alghamdi B., Al-Kadi M., Alkhayal N., Alhedaithy R., Al Mahdi M.J. (2020). Intranasal lobular capillary hemangioma: a series of five cases. Respir. Med. Case. Rep..

[9] Sarwal P., Pyogenic Granuloma Lapumnuaypol K. (2023). http://www.ncbi.nlm.nih.gov/books/NBK556077/.

[10] Pagliai K.A., Cohen B.A. (2004). Pyogenic granuloma in children. Pediatr. Dermatol..

[11] Karagama Y.G., Howarth K., Steel P.R.M., Spencer M.G. (2002). Lobular capillary haemangioma of the nasal vestibule: a rare entity. Int. J. Pediatr. Otorhinolaryngol..

[12] Derkenne R., Coulet O., Varoquaux A., de Biasi C., Tomasi M. (2012). Nasal cavity lobular capillary hemangioma due to insect sting. Eur. Ann. Otorhinolaryngol. Head Neck Dis..

[13] Virbalas J.M., Bent J.P., Parikh S.R. (2012). Pediatric nasal lobular capillary hemangioma. Case Rep. Med..

[14] Lee G., Suh K., Lee Y., Kang I. (2012). CT findings in two cases of lobular capillary haemangioma of the nasal cavity: focusing on the enhancement pattern. Dentomaxillofac. Radiol..

[15] Lee D.G., Lee S.K., Chang H.W. (2010). CT features of lobular capillary hemangioma of the nasal cavity. AJNR Am. J. Neuroradiol..

[16] Yang B.T., Li S.P., Wang Y.Z., Dong J.Y., Wang Z.C. (2013). Routine and dynamic MR imaging study of lobular capillary hemangioma of the nasal cavity with comparison to inverting papilloma. AJNR Am. J. Neuroradiol..

[17] Chi T.H., Yuan C.H., Chien S.T. (2014). Lobular capillary hemangioma of the nasal cavity: a retrospective study of 15 cases in Taiwan. Balkan Med. J..

[18] Ifeacho S.N., Caulfield H.M. (2011). A rare cause of paediatric epistaxis: lobular capillary haemangioma of the nasal cavity. BMJ Case Rep..

[19] Lin G., Bleier B. (2016). Surgical management of severe epistaxis. Otolaryngol. Clin. N. Am..

[20] Jafarzadeh H., Sanatkhani M., Mohtasham N. (2006). Oral pyogenic granuloma: a review. J. Oral Sci..

[21] Agha R.A., Franchi T., Sohrabi C., Mathew G., Kerwan A., SCARE Group (2020). The SCARE 2020 guideline: updating consensus Surgical CAse REport (SCARE) guidelines. Int. J. Surg..

